# DipSkmer: Reference-free population genomics with diploid genome skims

**DOI:** 10.1371/journal.pcbi.1014823

**Published:** 2026-10-13

**Authors:** Eduardo Charvel, Homère J. Alves Monteiro, Siavash Mirarab, Vineet Bafna

**Affiliations:** 1 Bioinformatics and Systems Biology Graduate Program, University of California San Diego, La Jolla, California, United States of America; 2 Center for Evolutionary Hologenomics, GLOBE Institute, University of Copenhagen, Copenhagen, Denmark; 3 Department of Electrical and Computer Engineering, University of California San Diego, La Jolla, California, United States of America; 4 Department of Computer Science & Engineering, University of California San Diego, La Jolla, California, United States of America; University of Helsinki: Helsingin Yliopisto, FINLAND

## Abstract

Ecologists and conservation biologists rely on genetic diversity as an essential biodiversity variable (EBV) used to track population health and dynamics, and utilize the population-scaled mutation parameter θ as a key metric of diversity. While whole-genome sequencing (WGS) is increasingly affordable, it will be considerable time before the full diversity of life is represented by high-quality assembled genomes; even then, constant monitoring will still require repeated sampling across time and space. In contrast, genome skimming (low-coverage, short-read wgs) is highly cost-effective but challenging to analyze because the coverage is too low for assembly and reliable error correction. Mature methods, such as Mash, exist for estimating pairwise genomic distances based on the Jaccard similarity of *k*-mer sets computed using sketching techniques. Some, such as Skmer, additionally model the impacts of low coverage. These methods have been successfully applied to assembly-free species identification and phylogenetics; however, their use in population genetics has been limited. Among other limitations, these methods implicitly treat genomes as haploid, and heterozygosity confounds estimates of genomic distance for diploid organisms. In this paper, we address this problem through several technical advances. First, we use coalescent theory to mathematically derive how the Jaccard index between the *k*-mer sets of two diploid samples changes with θ under a neutral Kingman’s coalescent model. Next, we model the impact of coverage and sequencing error to derive an estimate of θ from the Jaccard index, in addition to several auxiliary variables, which we also estimate from the genome skims. The resulting method, DipSkmer, enables more accurate estimates of coverage, sequencing error, and θ for diploid samples. Analyses of both simulated and empirical datasets show that for diploids and low distances (e.g., < 2%), DipSkmer’s estimate outperforms existing alignment-free methods such as Mash and Skmer, and closely approximates ANGSD, a reference and alignment-based tool.

## Author summary

Repeated calculation of full-genome population genomic measurements for biodiversity monitoring across time and space remains expensive due to the need for high-coverage sequencing and genome assemblies. Genome skimming is a viable, low-coverage alternative for obtaining genomic distances, and alignment- and assembly-free methods exist for analyzing nuclear data from skimming data to estimate the distance between samples. However, existing methods fail to model within-sample heterozygosity, expected for diploid organisms. Given the dominance of diploidy among species of interest to ecologists, the implications of these simplifying assumptions warrant further study. Here, we present a mathematical model of the *k*-mer sets sampled from two diploid genomes under Kingman’s coalescent model. We use the model to develop DipSkmer, a *k*-mer-based, reference-free method for estimating genetic diversity that, unlike its predecessors, models within-sample heterozygosity. Benchmarking shows more accurate genomic diversity estimates compared to existing reference-free, genome-skimming methods and comparable performance to the popular high-coverage, reference-based method, ANGSD. Thus, DipSkmer enables accessible, less expensive population monitoring through genetic diversity estimates.

## 1 Introduction

Nucleotide diversity, measured as the fraction of positions in the genome that differ between two organisms, is widely used across biology. This distance measure is used in phylogenetics, microbiome analyses, comparative genomics, and population genetics as a simple measure of evolutionary divergence. Nucleotide diversity is highly heterogeneous across genomes because it is shaped by local mutation rate, recombination and linked selection [1]. Consequently, genome-wide estimates represent averages over substantial biological variation. Nevertheless, it is informative and reflects the combined effects of mutation, drift, selection and demographic history. Therefore, it is among the Essential Biodiversity Variables (EBV) used extensively by ecologists and conservation biologists to monitor the health of species [2]. Species with dwindling populations often (but not always [3,4]) suffer from reduced genetic diversity [5], which can lead to loss of fitness [6]. Monitoring the health of species, which is increasingly important as biodiversity continues to face unprecedented levels of strain [7,8], requires not just a one-time estimate of diversity but continuous monitoring across spatial and temporal gradients. Such a goal becomes more practical if we have access to low-cost tools for monitoring.

When assembled genomes are available, genetic diversity can be measured by calling genotypes. While even a single diploid sample with high enough coverage can suffice for assembly *and* obtaining an estimate of genetic diversity [9], a single measurement will not capture temporal and spatial variation [10]. Even if an assembled genome is available, repeated sampling across time and location at high coverage will be expensive. Furthermore, much of the biodiversity on earth is not yet represented with genomes, and conservation biologists cannot afford to wait until such sampling is done. The viable alternative of barcoding a single marker gene gives limited resolution at the species level [11,12].

An alternative that has been put forward [13] and used with some success [14–21] is genome skimming, which consists of sequencing a sample at low coverage without assembling the nuclear genome (mitogenome can be assembled, a method many skimming-based analyses use [22]). Skimming can have a low cost and can eliminate the need for assembly. The *k*-mer sketching methods can then be used to efficiently compute distances between sets of reads for species identification. In particular, Ondov et al. [23] developed an efficient approach to use min-hash [24] to estimate the Jaccard (*J*) index between the *k*-mer sets of two samples, and used a simple transformation noted earlier by Fan et al. [25] to translate the Jaccard index to nucleotide distances. Later, Sarmashghi et al. [26] showed how the translation between *J* and distance can also account for effects of low coverage and sequencing error by pairing it with an analysis of the *k*-mer frequency spectrum, and Charvel et al. [27] showed how to account for the impact of repeats. Many researchers have built on various hashing techniques for fast distance calculation [28–33]. All these methods are in principle relevant to monitoring genetic diversity due to the link between the nucleotide diversity and standard population genetic diversity metrics. In particular, for a pair of randomly sampled haploid chromosomes that coalesced *T*_2_ generations ago and accumulated mutations at a constant rate μ per generation, the expected nucleotide diversity is


$$\begin{array}{c} \pi =2\mu 𝔼[T_{2}]\;. \end{array}$$ (1)

Under simplifying assumptions of Kingman’s coalescence with a fixed population size $N_{e}$ haploid individuals, $𝔼[T_{2}]=2N_{e},$ connecting π to population scaled mutation rate, θ.

Existing *k*-mer-based methods make simplifying assumptions that ignore many aspects of genome evolution (e.g., repeat structure, paralogy, and insertions and deletions) and focus on substitutions. Even for substitutions, they have largely overlooked the complexity introduced by within-sample heterozygosity; effectively, they rely on the haploid-based Eq (1) to translate between nucleotide and genetic diversity. These methods fail to account for changes between the two alleles present in a single sample of a diploid organism, and instead model evolutionary changes on one chromosome. Such a model is suitable for application to microbial species, but insufficient for diploid organisms. Ignoring within-sample heterozygosity may be inconsequential when comparing samples from different species (e.g., for species identification), but it is important for population genetic analyses. Our goal is to provide an estimator that accounts for heterozygosity, which requires considering four haploid genomes.

Let $S_{n}$ denote the number of segregating sites among *n* genomes. Going backward in time, and assuming no recombination, the *n* haplotypes will coalesce to n−1, then to n−2, and eventually to a single haplotype. Let $T_{k}$ denote the time to coalesce from *k* to k−1 haplotypes. Assuming infinite sites and fixed mutation rate μ,


$$\begin{array}{c} 𝔼[S_{n}]=\mu \sum_{k=2}^{n}k𝔼[T_{k}], \end{array}$$

Under a neutral, randomly mating, constant-sized population model with $N_{e}$ diploid individuals (i.e., Kingman’s coalescence), we have $𝔼[T_{k}]=4N_{e}/(k(k-1)),$ which gives us:


$$\begin{array}{c} \theta :=4\mu N_{e}=\frac{𝔼[S_{n}]}{\sum_{i=1}^{n-1}1/i}=\frac{\mu \sum_{k=2}^{n}k𝔼[T_{k}]}{\sum_{i=1}^{n-1}1/i}\;. \end{array}$$ (2)

We will use this equation to model a pair of diploid individuals, by choosing *n* = 4 chromosomes paired *randomly* into two diploids, and refer to θ as *genetic diversity* henceforth. When the coalescence scenarios are more complex than Kingman’s (varying population size, sub-population structure, etc.), $N_{e}$ diverges from the census population size, but the “effective” θ estimated using Eq (2) remains a useful summary metric of genetic diversity, reflecting the combined effects of mutation, drift, selection, and demographic history.

We address the following specific question: *Given reads generated from two diploid individuals sampled from a population evolving under a model consistent with Kingman’s coalescent process, how does the Jaccard index between their k-mer sets relate to* θ, *and how does Jaccard change as coverage drops and sequencing errors are added?*

Our approach first uses coalescent theory to characterize the Jaccard index with respect to θ for diploid genomes (Methods 4.2). It then models how Jaccard changes given low-coverage genome skims (Methods 4.3), and how to estimate parameters needed for Jaccard computation (Methods 4.4). We use these derivations to develop DipSkmer (Diploid Skmer), which takes as input two sets of reads from closely related diploid organisms and outputs the effective population-scaled mutation rate θ (assuming Kingman’s coalescence). In extensive simulations, DipSkmer shows substantially higher accuracy compared to Mash and Skmer for diploid samples. On real data, we show that DipSkmer’s θ estimates correlate strongly with those from high-coverage data mapped to a reference genome.

## 2 Results

### 2.1 Diploidy alters the Jaccard index’s relationship with genetic diversity

When comparing *k*-mers between two diploid genome skimming samples, each sample carries its own set of heterozygous and homozygous *k*-mers, which are in turn homozygous or heterozygous across both samples, thereby creating a complex relationship between within-sample heterozygosity and *k*-mer set similarity (Fig 1A). At the individual *k*-mer level, it is hard to know whether it is heterozygous in each sample. However, by assuming Kingman’s coalescent model, we can model the expected number of *k*-mers following particular evolutionary patterns, and thereby characterize how genetic diversity shapes the Jaccard Index. In DipSkmer, we use a coalescent model to capture how *k*-mers, treated as independent haplotype blocks, can have distinct evolutionary histories (Fig 1B). Given the resulting *k*-mer alleles, we then model how they are represented in the Jaccard Index (Fig 1C), and finally incorporate a coverage and error model to account for missing and erroneous *k*-mers within the diploid framework (Fig 1D), yielding a final estimate of θ (Fig 1E).

**Fig 1:**
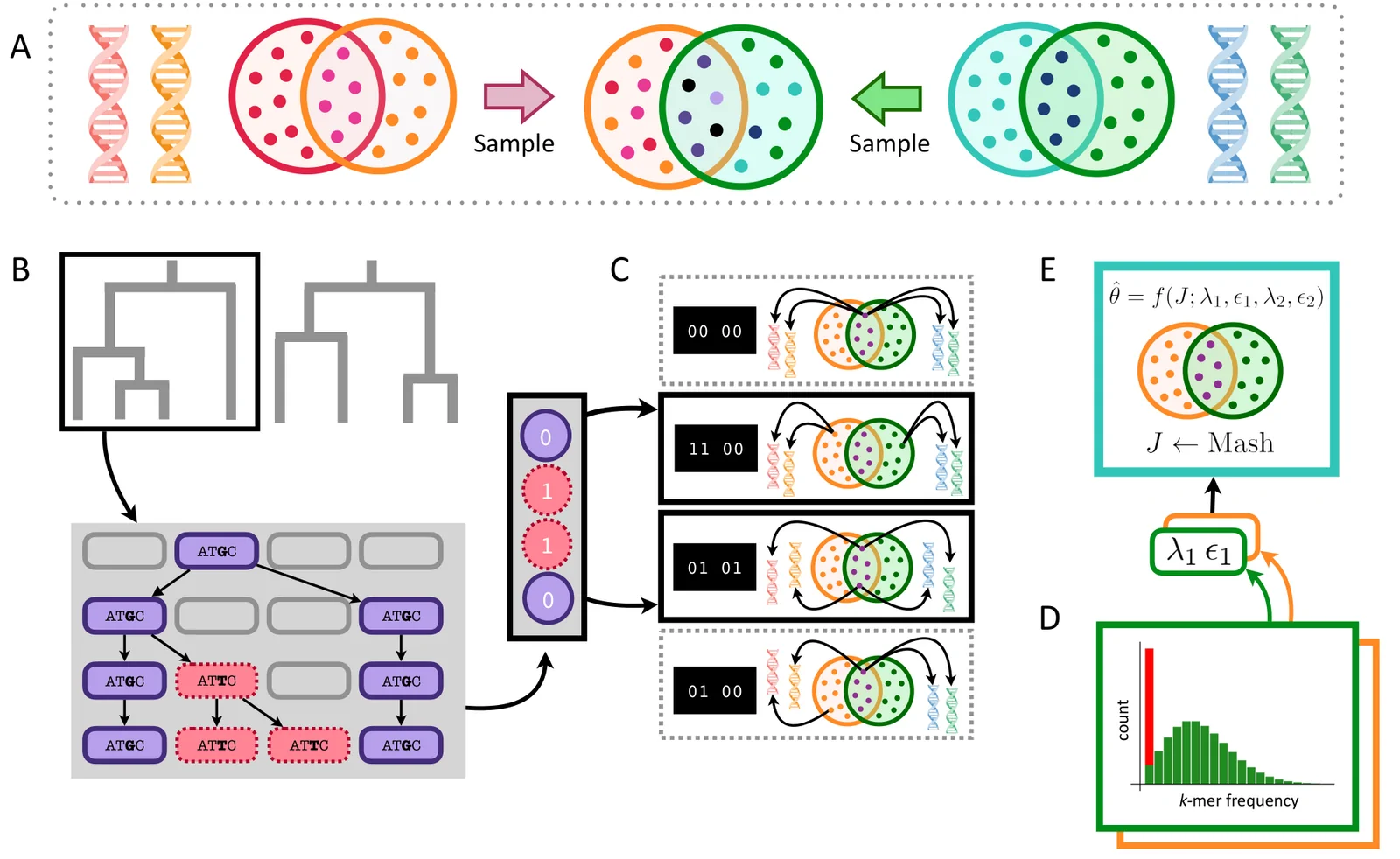
DipSkmer estimates genetic diversity between two diploid genomes, using only low coverage, error-prone, unassembled genomic data. **A)** The Jaccard index computed from genomes of two diploid organisms measures similarity between two pairs of haploid chromosomes, where each sample contains its own homozygous and heterozygous *k*-mers. **B)** We can model the possible coalescence scenarios of the four independent *k*-mers obtained from each position of two diploid genomes. For each topology, depending on the branches where mutations are introduced, *k*-mers may appear in one or both samples with computable probabilities (see Fig 6). **C)** The alleles resulting from these mutations are then independently sorted into each haploid, leading to 0, 1, or 2 shared *k*-mers per position. **D)** We incorporate a model to estimate coverage (λ) and error (ϵ) based on *k*-mer frequency spectrum of the sample; erroneous *k*-mers (red) tend to be less frequent, whereas error-free *k*-mers tend to follow a Poisson distribution (green). **E)** We derive the expected numbers of *k*-mers shared in scenarios shown in (D) to design an estimator of genetic diversity (Eqs (4) and (5)) given an observed Jaccard index and estimates of coverage and error. (DNA molecule graphic adapted from NIAID Visual & Medical Arts [34]).

**Estimating** θ **between diploid samples.** Under a diploid model, *k*-mer loci will occur twice in a DNA sample, making it so that each locus can weight the Jaccard differently with different configurations of heterozygous *k*-mers (Fig 1C). DipSkmer’s model (Methods 4.2: Extending to two diploid individuals (*n* = 4)) assumes that all haplotypes are sampled from a randomly mating population, such that coalescence between the paternal and maternal alleles within a sample is identically distributed to coalescence between alleles from different individuals. Each set of haplotypes can then be traced back to one of two topologies, each with its own probability of occurrence (Fig 1B). Under the infinite sites assumption, we model how mutations may fall on each topology (Fig 1C) and assign a random permutation of haplotypes to the topology leaves (Fig 1D). We then analyze the expected number of *k*-mers in the intersection (*I*) and union (*U*) at a single genomic position. Since different substitution patterns contribute differently to the union and intersection, enumerating all scenarios yields: $𝔼[I]=1+\frac{1}{11}(1-Q)$ and 𝔼[U]=2−Q. where *Q* is defined as the probability that no mutation happens on a lineage and is approximately $(1-\theta {)}^{k}$. Using the method of ratios from Mash/Skmer and solving for *Q*, we obtain:


$$\begin{array}{c} J=\frac{1+\frac{1}{11}(1-Q)}{2-Q}⟹Q=\frac{2J-\frac{12}{11}}{1+J-\frac{12}{11}}⟹\hat{\theta }=1-{\left(\frac{2J-\frac{12}{11}}{1+J-\frac{12}{11}}\right)}^{\frac{1}{k}}. \end{array}$$

**Diploid parameter estimation.** To the diploid Jaccard model, we introduce a low-coverage model (Methods 4.3: Modeling low coverage and errors) that estimates the probability of a locus contributing an observed *k*-mer to the union and intersection of the Jaccard Index, given estimates of *k*-mer coverage (λ) and sequencing error rate (ϵ) (Fig 1E). To estimate these sequencing parameters, we derive a new set of equations that account for how within-sample heterozygous *k*-mers affect the distribution of observed *k*-mer counts (Methods 4.4: Parameter estimation for diploid skims). Specifically, we take the ratio of consecutive *k*-mer count bins to obtain an error-free *k*-mer coverage estimate (ξ), which yields an internal, per-sample estimate of θ and, in turn, a θ-informed estimate of λ and sequencing error rate ϵ.

### 2.2 Simulation results

In order to test DipSkmer’s accuracy, we use msprime [35] to simulate diploid populations of different base genomes as explicitly defined in Experimental setup. By modulating effective population size, simulated samples result in having different genetic diversity values (θ∈{0.001,0.003,0.006,0.009,0.012}). To test whether DipSkmer can also estimate the divergence between populations, we perform separate msprime simulations that lead to populations having different levels of genetic divergence (0.03125, 0.0625, 0.125, 0.25, 1) in coalescent units ($\tau /4N_{e}$). Note that these simulations violate the random mating assumptions of DipSkmer, and thus, test the robustness of the method (especially at higher divergence levels). Moreover, the presence of repeated *k*-mers in genomes violates DipSkmer assumptions; we focus on three base genomes with *k*-mer uniqueness above 0.7 in the main results. To test the degradation of performance with extremely high levels of repeats, we separately examine three extra genomes (Experimental setup). After generating genomes, we use ART [36] to simulate reads for each simulation at varying coverages (c∈{0.5×,1×,2×,4×,8×}).

We compare DipSkmer to its predecessor alignment-free methods, Skmer (which effectively assumes haploid input) and Mash (which additionally ignores low coverage). We also compare to ReSkmer, which like Skmer ignores heterozygosity but unlike DipSkmer models repeats in the genome. We compare θ estimates using these methods to the true θ (the percentage of substituted positions in genome). We measure error as $\frac{\left(\hat{\theta }-\theta \right)}{\theta }$, averaged over all replicates, where $\hat{\theta }$ is the estimated pairwise distance and θ is the true pairwise distance. We also report mean absolute error (MAE), defined as $\frac{|\hat{\theta }-\theta |}{\theta }$. For population differentiation experiments, we estimate $D_{xy}$ (average θ between pairs of samples taken from different populations) and Weir and Cockerham’s $F_{st}=1-\frac{\theta_{x}+\theta_{y}}{2D_{xy}}$ [37], comparing estimated values to the ground truth, calculated by counting substitutions.

#### 2.2.1 DipSkmer provides better θ estimates with multiple iterations.

Before comparing DipSkmer to other methods, we first compare per-sample and per-pair estimates of θ. Recall that we use *k*-mer frequency spectrum to obtain a θ for each sample used in coverage calculations (Methods 4.4), in addition to the pairwise estimate of θ based on Jaccard. In simulated datasets, pairwise Jaccard-based estimates of θ are far more accurate than within-sample frequency-spectrum-based estimates, especially with low coverage (Fig A in S1 Text). Based on this observation, we propose an iterative approach to estimate θ using the within-sample method, estimate coverage, estimate pairwise θ, and use this new θ to obtain better coverage, iterating this process several times. DipSkmer’s average error reduces from 46% with the within-sample method to 29% error in the third pass of the iterative approach (Fig B in S1 Text). With the exception of high coverages (4× and 8×), the third iteration of DipSkmer is more accurate than using within-sample estimates from Eq (9). On empirical data, correlations stay stable across passes (Fig C in S1 Text). However, going from one pass to three passes tends to help accuracy in cases where the true θ is much lower than the starting $\theta^{*}$ used in the first pass (0.005). While it is not universally the best, the third pass is used in all subsequent DipSkmer results.

#### 2.2.2 DipSkmer θ estimates are more accurate than haploid methods.

Across most coverages, populations, and parental genomes, DipSkmer provides substantial improvements compared to ReSkmer-ref, Skmer, and Mash (Fig 2 and Table A in S1 Text). Overall, DipSkmer has the smallest MAE (25%), which is a substantial improvement compared to ReSkmer (67%), Skmer (86%) and Mash (619%) (Fig D in S1 Text). Comparing coverage levels, DipSkmer obtains its highest accuracy at 2× (with 17% MAE) where it provides substantial improvements over ReSkmer-ref (57%), Skmer (81%) and Mash (476%). Only at 8×, the highest simulated coverage, does Skmer achieve comparable accuracy (19% MAE) to DipSkmer. Mash has the best accuracy at 8× coverage when θ is ≥0.009, but is otherwise inaccurate; it has very high error when coverage is below 4× or when estimating small distances (θ<0.005); even at 8× coverage, it has an error of 190% for these small distances.

**Fig 2:**
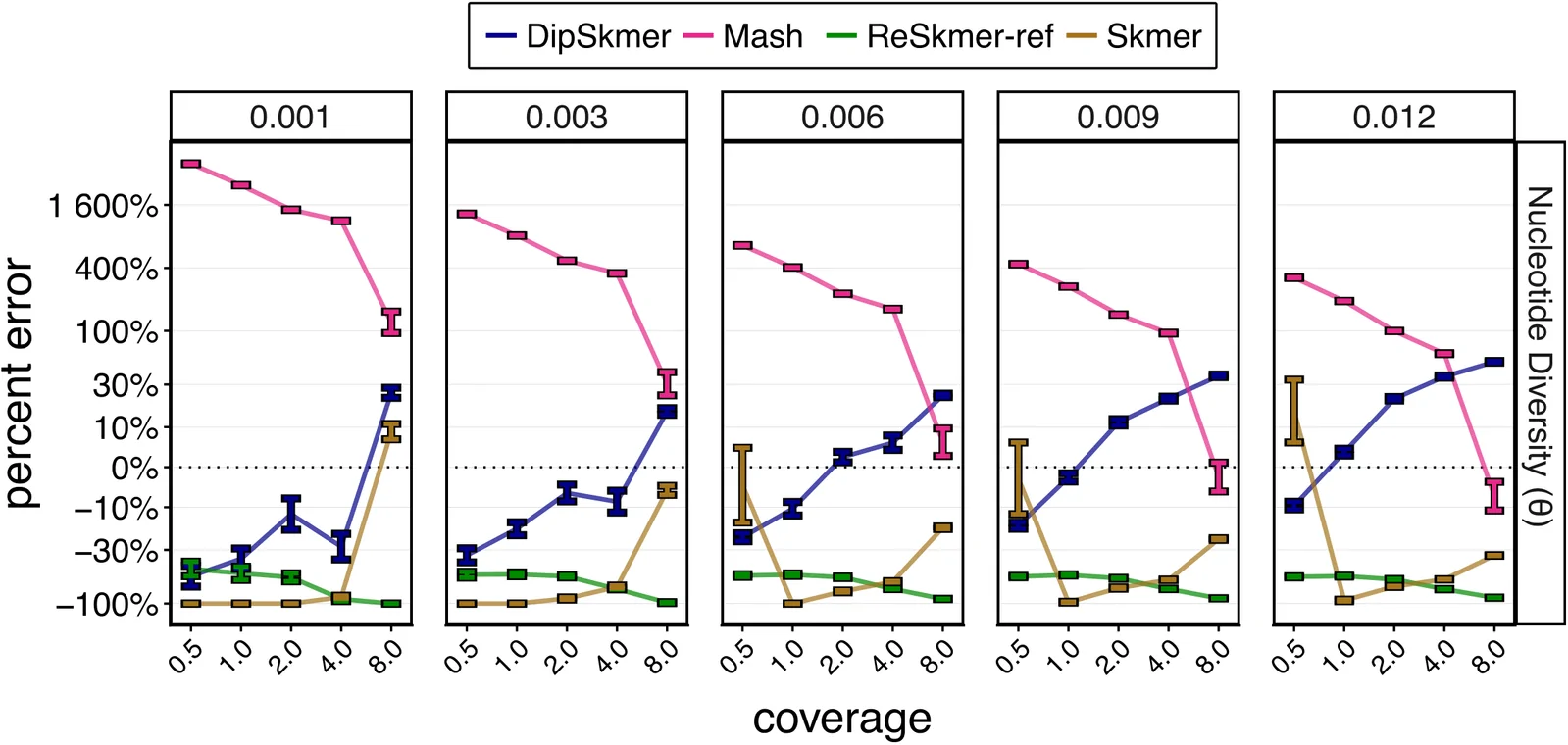
Accuracy of genetic diversity (θ) estimates on simulated data. Percent error ($\frac{\left(\hat{\theta }-\theta \right)}{\theta }$) plotted against sequencing coverage. Panels correspond to the true θ value for each simulated population size. Bars show the range of error across 5 replicates of three genomes. *Note:* error of -100% means the method estimated $\hat{\theta }$ = 0.

When comparing methods at very low coverages (0.5× and 1×) DipSkmer is far more accurate than other methods. At these coverages, Skmer consistently outputs an estimate of zero when the simulated θ≤0.003. ReSkmer-ref is as accurate as DipSkmer for θ≤0.003, but not for higher θ. Examining each of the three parental genomes separately (Figs D and E in S1 Text), we observe that DipSkmer is especially accurate in the least repetitive genome we simulate (leech) with a MAE of 20% (compared to ReSkmer-ref’s 79%, Skmer’s 70%, and Mash’s 626%). DipSkmer’s error for the two more repetitive genomes only increases to 28%, while Skmer error rises to 97%. Among these moderately repetitive genomes (UR > 0.7), ReSkmer-ref becomes more accurate as repeat content increases, but remains less accurate than DipSkmer in most situations. However, for extremely repetitive genomes (UR∈{0.6;0.5;0.4}) and low θ (θ=0.001−0.003), ReSkmer-ref becomes more accurate than DipSkmer (Fig F in S1 Text), which often produces 0 distance. Even for these genomes, DipSkmer is competitive with ReSkmer at higher θ values, outperforming ReSkmer at low coverages.

We also test how accurately DipSkmer estimates sequencing parameters compared to Skmer and ReSkmer (Fig G in S1 Text). In terms of coverage, error rate, and genome length, DipSkmer clearly outperforms Skmer. There is a large improvement in sequencing error estimates at 0.5× and 1× coverage, with more modest gains for higher coverages. The estimated coverage and genome length similarly improve the most at lower coverages but gain very little at higher coverages. ReSkmer-ref understandably outperforms DipSkmer in genome length and coverage estimations, given we are providing an error-free reference genome. In spite of not being reference-based, DipSkmer obtains more accurate sequencing error estimates than ReSkmer-ref when coverage ≥2×. Despite these improvements, which are likely due to modeling within-sample θ in repeat-spectrum analyses, some overestimation of coverage and thus underestimation of genome length remain, perhaps due to inaccuracies in estimating within-sample θ (Fig A in S1 Text).

#### 2.2.3 DipSkmer accurately measures population divergence with $D_{xy}$ and $F_{ST}$.

We next measure DipSkmer’s ability to measure divergence between populations in simulated data, starting with the fixation index, Weir and Cockerham $F_{ST}$ (Fig 3). DipSkmer and Skmer both overestimate $F_{ST}$, but DipSkmer has a lower error in most conditions, with some exceptions among high coverage and high divergences (coalescent unit 1 divergence, which often corresponds two different species). Notably, with lower coverage, DipSkmer is much more accurate than Skmer, and its accuracy is highest at around 1–2× coverage. Mash has the opposite error, underestimating $F_{st}$ in all cases with coverage below 8×. Mash essentially fails to detect differentiation between populations when coverage is below 4×. Conversely, Mash is very accurate when the population differentiation is at the speciation level (1 coalescent unit) and coverage is high (i.e., conditions it is designed for); in these conditions, which strongly break DipSkmer’s assumptions of random coalescence, DipSkmer fails to detect differentiation at 4–8× coverage. Nevertheless, DipSkmer produces accurate estimates at 0.5–2× coverage despite strong model misspecification. We also test two more definitions of $F_{ST}$, Nei’s and Slatkin’s, and observe similar results (Fig H in S1 Text). Repeats play a role in all methods’ $F_{st}$ estimation accuracy (Fig I in S1 Text); DipSkmer achieves its highest accuracy in low repeat genomes and becomes less accurate in higher repeat genomes. However, the relative accuracy of methods remained similar across repeat levels.

**Fig 3:**
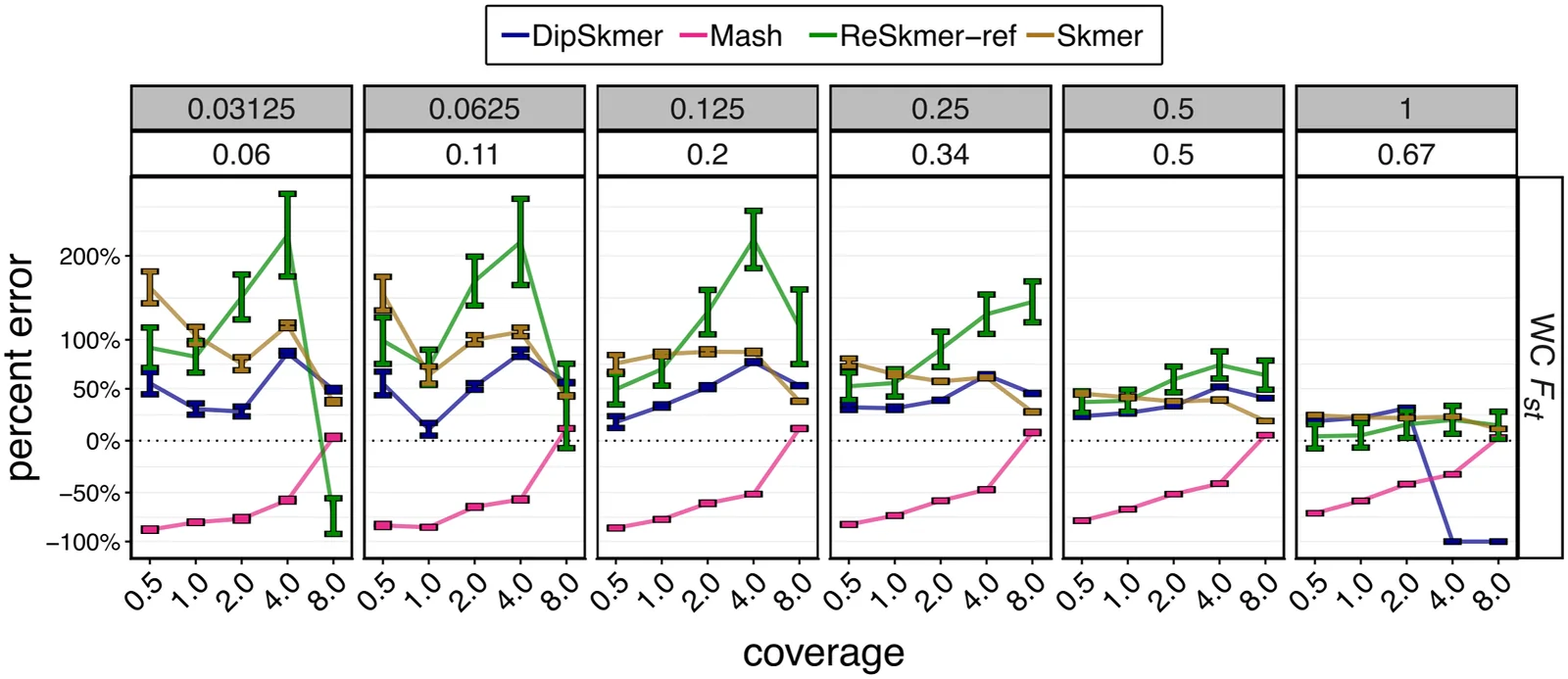
Accuracy of fixation index ($F_{st}$) on simulated data. We show results for Weir and Cockerham $F_{st}$ across two simulated populations at a constant population size ($N_{e}=5\times 10^{5}$). Panels correspond to different levels of divergence in coalescent units (gray strip) with the corresponding mean true WC $F_{st}$ shown below.

Another measure of divergence we test is the average distance between samples of different populations, $D_{xy}$ (Fig J in S1 Text). For $D_{xy}$, DipSkmer is the most accurate method in most conditions, with some exceptions when divergence is very high or the coverage is 8×. Skmer and Mash both perform better than DipSkmer at 8× coverages. ReSkmer-ref has a similar performance to Skmer, but becomes more inaccurate as coverage increases. The $D_{xy}$ metric clearly shows the impact of population divergence on DipSkmer: As population divergence increases (breaking random coalescence assumptions), the accuracy of $D_{xy}$ declines steadily, especially above 0.25 coalescent units divergence. In contrast to $F_{st}$ and θ, repetitiveness does not affect $D_{xy}$ estimation accuracy much. DipSkmer’s accuracy stays around 31%-32% regardless of repeat level (Fig J in S1 Text).

### 2.3 Biological results

To test DipSkmer on empirical data, since true θ values are not known, we compare *k*-mer-based methods to previously published [38] θ estimates obtained by ANGSD [39], which is a widely used method based on read mapping, genotyping, and computing Site Frequency Spectrum (SFS). The dataset is composed of 6 species and 12 populations (Table B in S1 Text). To get measures of correctness of our genetic diversity estimates, we measure the difference between θ values and the Pearson correlation of our estimates of θ to those produced by ANGSD. We follow a published protocol [40] for the preparation of reads (described in Methods). We then subsample all genome skims to target coverages c∈{0.5×,1×,2×,4×,6×,8×}.

#### 2.3.1 DipSkmer closely matches ANGSD θ estimates in empirical datasets.

On the biological dataset, to gauge DipSkmer’s ability to quantify relative changes in genetic diversity, we first test how well DipSkmer correlates with ANGSD estimates of θ (Fig 4A). According to the Pearson correlation coefficient (*R*), DipSkmer has the highest correlation averaged across coverages and populations (*R* = 0.92), followed closely by Skmer (*R* = 0.9) and ReSkmer-noref (*R* = 0.9), all of which perform far better than Mash (*R* = 0.55). Mash is essentially uncorrelated at lower coverages, starts to have some correlation at 2×, and matches other methods at 6× or higher. DipSkmer, in contrast, has high correlation coefficients at all coverage levels, with minimal change across coverage.

**Fig 4:**
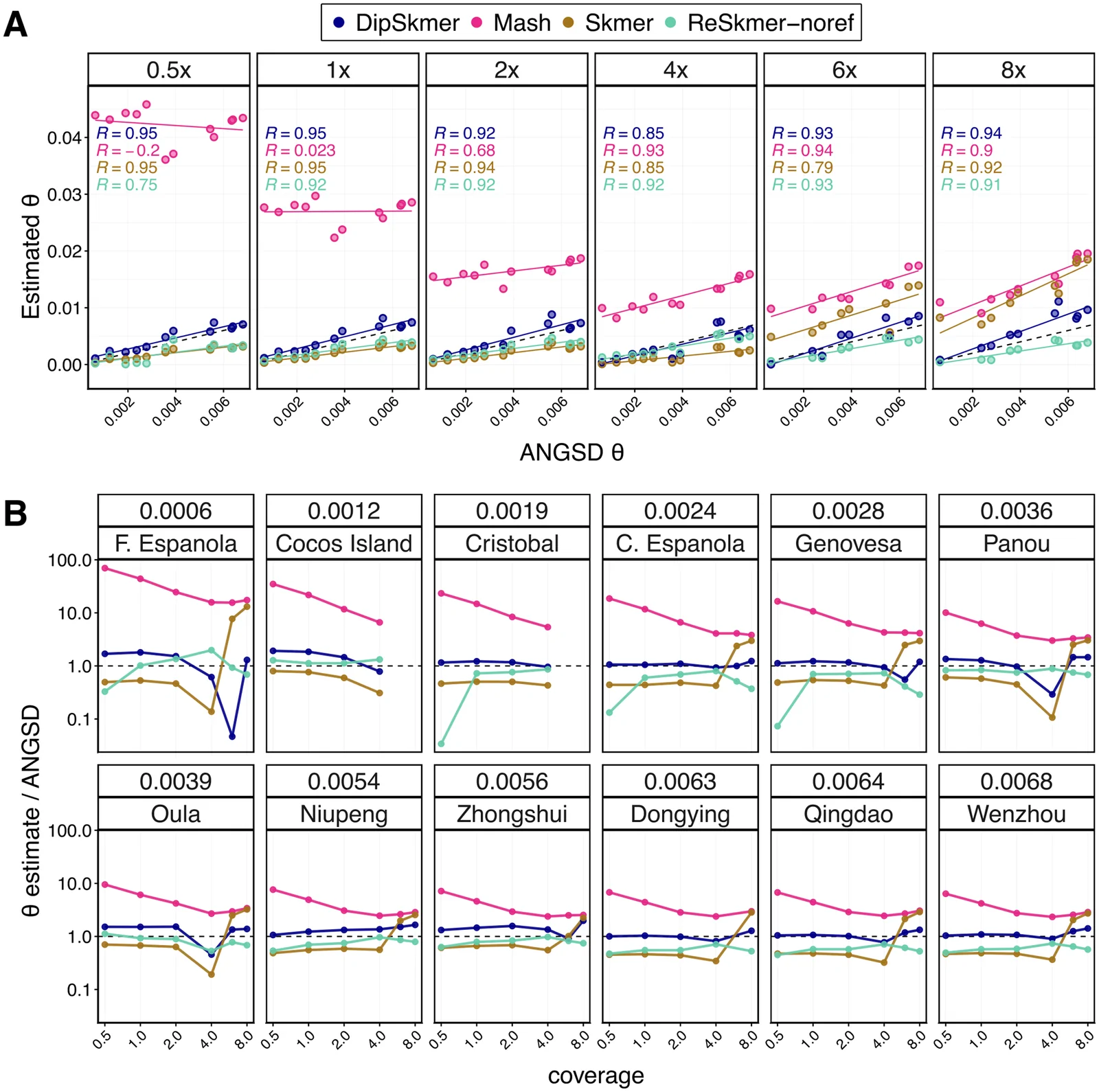
Correlations and accuracy in empirical datasets. **A)** Correlation of DipSkmer, ReSkmer-noref, Skmer, and Mash estimates of θ (*y*-axis) to ANGSD (*x*-axis). Each panel corresponds to a different level of coverage. Pearson correlation coefficients (R) are found in the top left corner of each panel. **B)** Ratio (*y*-axis) between DipSkmer, Skmer, and Mash θ estimates and ANGSD θ across different coverages (*x*-axis). Panels represent different populations, with population name and corresponding ANGSD estimate on the top strips.

Beyond correlations, DipSkmer also closely matches ANGSD estimates of θ, in contrast to Mash, which systematically overestimates, and Skmer, which systematically underestimates ANGSD (Fig 4B). ReSkmer-noref is competitive with DipSkmer, except at 0.5× coverage, where ReSkmer underestimates θ in some populations (see C. Espanola, Cristobal, and Genovesa). Across all populations and coverage, DipSkmer produces the most similar distances to ANGSD judged by MAE (28%, pooled across all comparisons) compared to ReSkmer-noref (41%), Skmer (83%), and Mash (738%); this pattern remains correct for each coverage level (Table A in S1 Text). Overall, the impact of coverage on DipSkmer is relatively low, with MAE ranging between 22% and 40%, with the peak accuracy achieved at 2× coverage. Meanwhile, ReSkmer-noref achieves its peak accuracy at 4× coverage (30% MAE), where it is nevertheless outperformed by DipSkmer (26%).

For *Sillago sinica* populations (Dongying, Qingdao, and Wenzhou) we test the similarity of reference-free estimates of θ between pairs of samples to those produced by an ANGSD-produced identity by state (IBS) matrix. IBS measures a normalized genetic distance between aligned loci given reference genomes and high coverage samples – which DipSkmer does not require. Again, DipSkmer produces the most similar estimates to ANGSD (Fig K in S1 Text), with a mean percent difference of 12% across all coverages. The two next most accurate methods have similar percent error: ReSkmer (30% for both noref and ref) and Skmer (43%). There is no substantial change in error between coverages, except for Mash whose error decreases from 687% (0.5×) to 237% (6×). Notably, even though coverage is relatively high, Mash remains highly inaccurate.

#### 2.3.2 DipSkmer best approximates ANGSD estimates of *Sillago sinica* population structure.

To test how DipSkmer performs in the presence of population structure, we take three populations from the same species (*Sillago sinica*) and look at two metrics for comparison: $F_{st}$ and Principal Coordinates Analysis (PCoA). We perform PCoA on identity by state (IBS) matrices produced by ANGSD as well as those produced by reference-free methods to project distances onto Euclidean space, followed by Procrustes to align the projections to enable comparison across methods.

Projections based on DipSkmer distances are remarkably close to those based on ANGSD (Fig 5A). In fact, in most cases, ANGSD projections are within the confidence intervals for DipSkmer (obtained using subsampling reads, see Methods). Skmer and ReSkmer-ref also capture the broad patterns but show lower similarity in their projections to those of ANGSD, in particular for four outlier Dongying samples. Judged by the alignment of points to ANGSD, as measured by the *t*_0_ similarity score (see Methods), DipSkmer is closest to ANGSD across all coverages and achieves very high similarity (*t*_0_ = 0.821) for 2× – 6× (Fig 5B). All methods have lower *t*_0_ values at coverage ≤1×, reach their peak at 2×, with little change at higher coverages.

**Fig 5:**
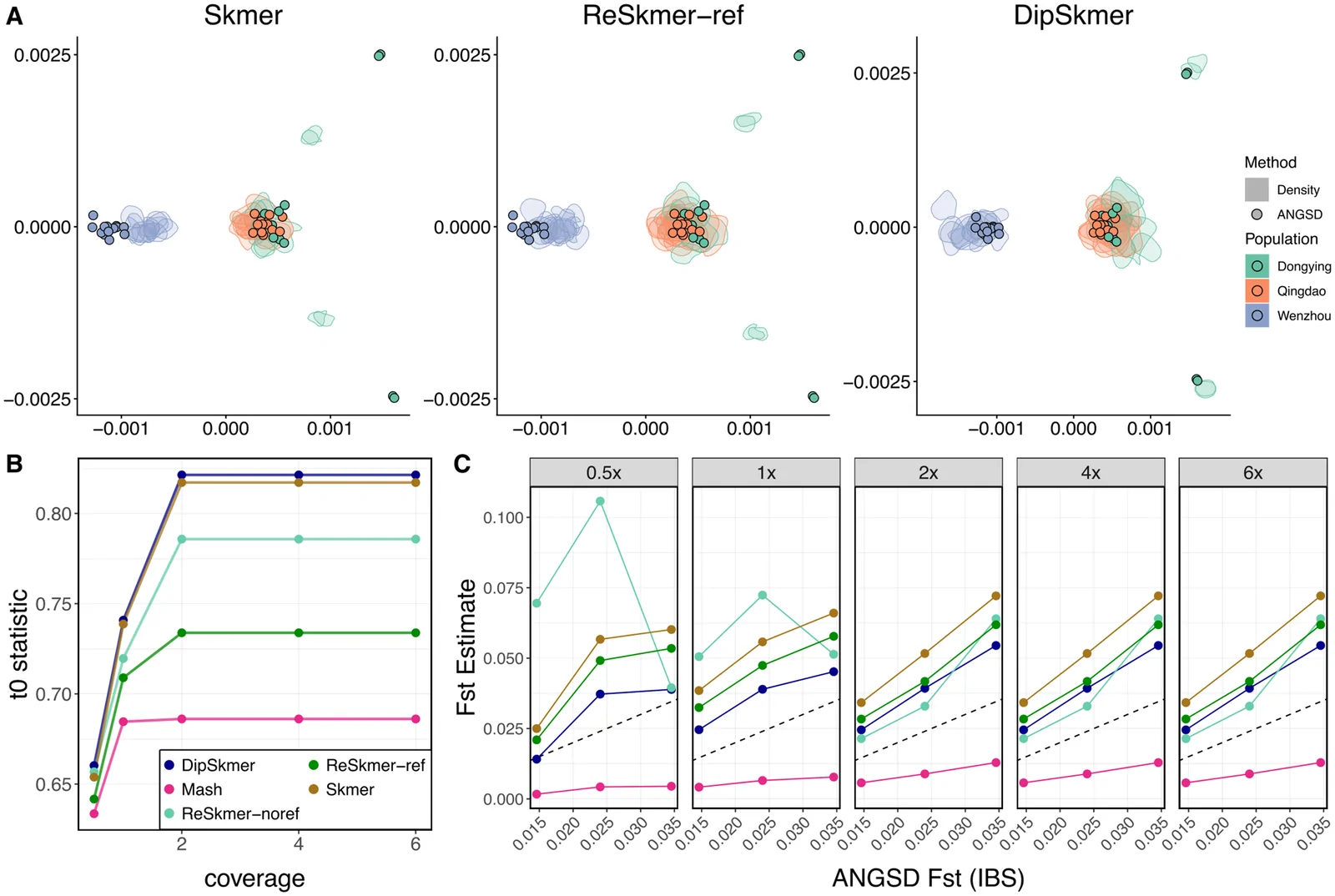
*Sillago sinica* population structure: A) Similarity between PCoA produced by ANGSD and those produced by three different alignment-free methods. Colors represent three different *Sillago sinica* populations. Points show the coordinates of ANGSD PCoA results and contours show the location of the highest 33% density of data points for each sample using the corresponding method. **B)** Similarity in PCoA structure (*t*_0_) between PCoA produced by ANGSD and those produced by skimming methods. The *t*_0_ statistic ranges from 0 (dissimilar) to 1 (identical). **C)** Correlation plot between reference-free estimates of Weir and Cockerham $F_{st}$ (*y*-axis) and those produced using the ANGSD IBS matrix (*x-axis*). The dotted line denotes the identity line.

To test the accuracy of $F_{st}$ measurements across methods, we use the distances output by reference-free methods to compute Weir and Cockerham (WC) $F_{st}$ (see Methods) and compare those to WC $F_{st}$ calculated from ANGSD IBS matrices (Fig 5C). At 0.5× and 1×, DipSkmer produces the most similar estimates to ANGSD. However, at >1×, ReSkmer-noref produces slightly more similar estimates to ANGSD for two population pairs, Qingdao-Dongying and Wenzhou-Qingdao, followed by DipSkmer. Finally, we also compare our $F_{st}$ values to those produced by 2D-SFS analysis with ANGSD (Fig L in S1 Text). Here, again, DipSkmer is the most accurate among reference-free methods at low coverages (22% error when coverage <2×).

## 3 Discussion

Here we presented DipSkmer, a method that models diploidy’s effect on the Jaccard index of *k*-mer sets to produce accurate and robust estimates of population-scaled mutation rate θ from diploid samples. In both simulated and empirical datasets, DipSkmer outperformed other alignment-free, reference-free methods for diploid inputs. Additionally, it was able to estimate measures of population differentiation such as $F_{st}$ and $D_{xy}$ with high accuracy. Moreover, DipSkmer is fast, requiring only 5 minutes on average on our datasets for each sample (Table C in S1 Text), which is identical to Skmer, and not much higher than Mash, which needs one minute per sample.

Interpretation of DipSkmer’s results requires some care. While genetic diversity is an essential biodiversity variable for assessing population health, the relationship between the two is complex and far from linear [4], with some persistent populations maintaining low genetic diversity, and vice versa. The genetic diversity is highly heterogeneous across the genome because it is shaped by local mutation and recombination rates, as well as selection [1]. Across a wide range of species, the range of neutral diversity levels indicates an 800X variation, many orders of magnitude smaller than the variation in census population sizes [3]. Thus, the changes in census population size may not be accurately manifested as changes in effective population size measured by θ under Kingman’s coalescent. Despite limitations of genetic diversity as a measure, changes in contemporaneous genetic diversity and effective population size relative to historical values or across locations can provide valuable, if incomplete, information about the health of the species.

DipSkmer’s promise for practical biodiversity monitoring lies in providing estimates that highly correlate with the more laborious option of mapping reads to a reference genome (ANGSD). While these methods can handle low coverage data sampled across multiple individuals, they still require a genome to map to. Since DipSkmer eliminates the need for access to assembled genomes, it opens up the possibility for population monitoring using genome skims for less well-studied species. Moreover, it works well at low coverages, further reducing costs. Counterintuitively, DipSkmer was generally more accurate with 2× coverage than 4×. Appendix B in S1 Text provides some theoretical insights as to why this may be the case: Around 4× coverage, changing θ surprisingly has little to no effect on how many *k*-mers are shared between two samples, reducing sensitivity. In practice, when coverage is 3–5×, we suggest downsampling reads to 2×.

The theory we provide allows estimation of θ from a single sample using its *k*-mer repeat spectrum or using two samples by modeling Jaccard. Given the ability to estimate heterozygosity from a single sample, which others have also attempted [41], one may wonder why the Jaccard-based estimates were necessary. Our empirical results showed that within-sample estimates of θ are far less robust, especially with low-coverage data (Fig A in S1 Text). In contrast, with correct coalescent modeling, Jaccard distances between pairs of samples provide highly accurate estimates of θ. Thus, we advise practitioners to use the pairwise estimates rather than single-sample estimates. At the same time, we make the assumption that the two samples are drawn from a well mixed population and have the same level of within-sample heterozygosity. When the samples are drawn from distinct sub-populations with differing effective population sizes, this assumption may not hold. Our future work will therefore aim to estimate within species heterozygosity, and use it in estimating the between-sample divergence.

Pairwise estimates additionally enable comparing populations (e.g., $D_{xy}$ and $F_{st}$), which is not possible with single-sample *k*-mer statistics. While our theory was based on Kingman’s coalescent model with random mating, DipSkmer was empirically robust when applied to individuals from two divergent populations, including at very low coverage and low population divergence. In extreme simulated cases where population separation approached the speciation-level divergences (e.g., around 1 coalescence unit in simulations), the accuracy of DipSkmer distance estimates drops. Thus, the use of this model should clearly be restricted to comparisons within a species or very close species. When populations are highly diverged, the impacts of within-sample heterozygosity become negligible compared to phylogenetic divergence, making other methods such as ReSkmer, Skmer, or Mash more appropriate. Despite our empirical robustness to moderate levels of population structure, future research should explore how differing coalescence dynamics in demographic models with population isolation and migration [42,43] affect the Jaccard index in theory.

Just as DipSkmer is an extension of Skmer that models heterozygosity, the recent ReSkmer method is an extension that models repeats based on *k*-mer frequency spectrum, which can be computed using RESPECT [44]. Unlike DipSkmer, ReSkmer (and RESPECT) ignore the impacts of heterozygosity. One should ideally model both aspects, but analytical equations become intractable. While we leave it to future work to combine the two models, we can empirically ask which aspect has a higher impact – accounting for repeats or heterozygosity. Based on our results, overall, heterozygosity appears to have a greater impact on Jaccard than repeats for population genetic level distances, though this pattern is not universal. When repeat content is extremely high and one has access to accurate reference genomes to compute *k*-mer frequency spectrum, ReSkmer can be more accurate (Fig F in S1 Text). Our results motivate future attempts to combine repeat modeling with within-sample heterozygosity. Due to the complexity of the resulting equations, the combined analyses may require moving beyond analytical approaches and adopting machine learning, following some earlier attempts [45]. Such combined modeling may also enable us to move beyond diploid organisms and model polyploidy and mixed-ploidy.

## 4 Methods

### 4.1 Background: Jaccard index for two haploids within a coalescence framework

The known connections between Jaccard and θ can be arrived at by analyzing the coalescent on *n* = 2 haploid samples. Let each *k*-mer be considered a set of *k* independent characters. For haploids, the expected time to coalesce under Kingman’s coalescent model is $𝔼[t_{2}]=N_{e}$, and thus, the expected number of mutations on the lineage is $\Lambda :=2N_{e}\mu k=\theta k$. The number of mutations can be considered a Poisson process with parameter Λ; then, $Q:=\mathrm{Pr}[\text{no mutation on the lineage}]=e^{-\Lambda }≃(1-\theta {)}^{k}$. Thus, for each position of genomes of length *L*, with probability *Q*, a single *k*-mer is added to both the intersection (*I*) and the union (*U*) of *k*-mer sets. If there is at least a mutation (probability 1−Q), the position adds 0 *k*-mers to the intersection and 2 *k*-mers to the union. Thus, 𝔼[I]=QL and 𝔼[U]=L(2(1−Q)+Q). Using the (biased) ratio estimator, one can arrive at $J=\frac{Q}{2(1-Q)+Q}=\frac{Q}{2-Q}$, which, solved for θ would give [23,25] $\hat{\theta }=1-{\left(\frac{2J}{1+J}\right)}^{\frac{1}{k}}$.

These calculations fail at low coverage and ignore sequencing errors [26]. Define *L*: = length of haploid genome, *N*: = number of sampled base pairs, ℓ:= read length, $\lambda :=\frac{N}{L}\frac{\ell -k+1}{\ell }$ *k*-mer coverage. We assume each *k*-mer has probability $\rho :=(1-\epsilon {)}^{k}≃e^{-k\epsilon }$ of being error-free and let ξ:=λρ be the error-free coverage of *k*-mers. Sarmashghi et al. [26] showed that ϵ,λ can be estimated for each sample from their *k*-mer frequency profile. Assuming random errors, $\eta :=\mathrm{Pr}[\text{an arbitrary k-mer at a specific location is sampled without error}]=1-e^{-\xi }$. Assuming errors generate unique *k*-mers, ζ:=𝔼[the number of unique{k−mersgeneratedfromaposition}]=η+λ−ξ, where η captures the error-free *k*-mers and the other terms the erroneous ones. Sarmashghi et al. [26] showed that with those estimates at hand for both samples (denoted by subscript), the nucleotide diversity (also, θ) can be estimated using


$$\begin{array}{c} 1-{\left(\frac{2(\zeta_{1}L_{1}+\zeta_{2}L_{2})J}{\eta_{1}\eta_{2}(L_{1}+L_{2})(1+J)}\right)}^{1/k}\;. \end{array}$$ (3)

### 4.2 Extending to two diploid individuals (*n* = 4)

We now turn to the diploid case, where we assume *n* = 4 haploids are sampled from a randomly mating population, so that coalescence between parental and maternal alleles of a sample is identically distributed to alleles from different individuals. We start by analyzing genomic *k*-mers (i.e., all *k*-mers are sampled without error), then model the impacts of low coverage and sequencing errors. Recall that for diploids, $\theta :=4N_{e}\mu$.

Consider the coalescence history of each locus, which can take one of two shapes (Fig 6A), with the unbalanced case twice as likely as the balanced. Under Kingman’s coalescence [46], for each time epochs $t_{i}$ (i∈{2,3,4}) with *i* lineages, $𝔼[t_{i}]=\frac{2N_{e}}{i(i-1)/2}$. The expected total branch length is $𝔼[\sum_{i=2}^{4}it_{i}]=\sum_{i=2}^{4}\frac{4N_{e}}{i(i-1)}i=\frac{11}{6}4N_{e}$. Under the infinite sites assumption, the number of substitutions per *k*-mer falling on these genealogies is Poisson distributed with parameter $\Lambda :=\frac{11}{6}4N_{e}\mu k=\theta \frac{11}{6}k$ and $Q:=\mathrm{Pr}[\text{no mutation in lineage of }n=4\text{ leaves}]=e^{-\Lambda }≃(1-\theta {)}^{\frac{11}{6}k}$ (compare to haploids). With probability P:=1−Q, we have one or more substitutions in a *k*-mer. We only analyze the possibility of zero or one substitutions, essentially assuming that in the unlikely event that multiple substitutions occur, the ratio of the expected number of shared *k*-mers to all *k*-mers is well approximated by derivations for one substitution. Conditioned on having exactly one substitution, $p_{i}:=\mathrm{Pr}[\text{substitution falls on a specific lineage of epoch }t_{i}]=\frac{t_{i}}{\sum_{i}it_{i}}$, and thus, $p_{2}=\frac{3}{11},p_{3}=\frac{1}{11},p_{4}=\frac{1}{22}$.

**Fig 6:**
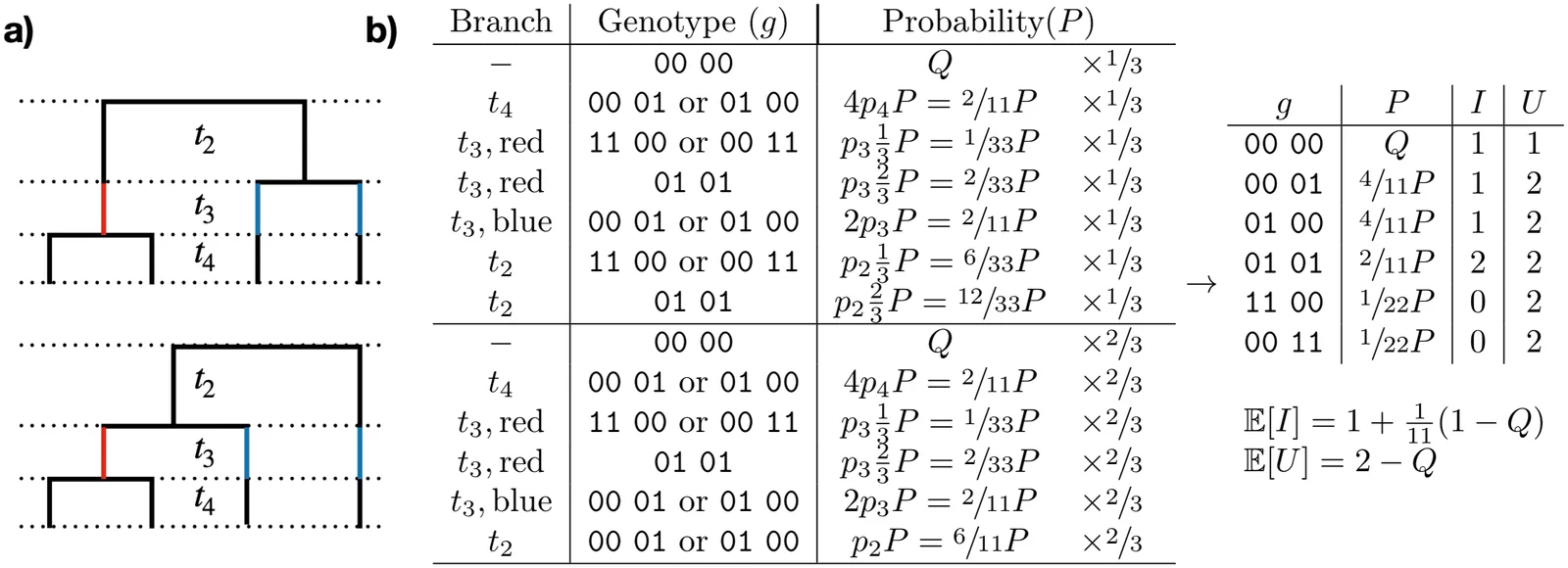
**a)** Possible coalescence topology shapes. Assignment of alleles to leaves is uniformly random. The epochs $t_{2}...t_{4}$ are marked. Note that in *t*_3_, the red branch can coalesce with either blue ones *t*o give the unbalanced tree, and thus, the probability of the unbalanced tree is $\frac{2}{3}$ versus $\frac{1}{3}$ for balanced. **b)** If a substitution occurs (probability P=1−Q), depending on which branch it falls on, it leads to different genotypes. Each genotype contributes 0–2 *k*-mers to the intersection (*I*) and 1 or 2 *k*-mers to the union *U* of the *k*-mer sets obtained from two diploid genomes.

We assign a random permutation of haplotypes to the leaves of the topologies and analyze the expected number of *k*-mers in the intersection (*I*) and union (*U*) for a single genomic position. Fig 6B summarizes analyses of both topologies. With probability *Q*, no substitutions occur, which leads to *I* = 1,*U* = 1. If any mutation occurs, *U* = 2. If it falls on any lineage in *t*_4_, the genotypes of the two individuals is some permutation of 00 01, which means, *I* = 1. If the mutation falls on the first lineage in *t*_3_, the two lineages carrying the mutation may belong to the same individual (this happens in 8 out of 24 $=\frac{1}{3}$ of all permutations), resulting in *I* = 0, or they may belong to different individuals, leading to *I* = 2. If the mutation falls on other lineages of *t*_3_, then *I* = 1. The *t*_2_ epoch depends on the *t*opology, where the balanced *t*ree has the same scenarios as *t*_3_, leading to *I* = 0 or (twice as likely) *I* = 2. For unbalanced, regardless of the permu*t*ation, both samples will have one of each allele, leading to *I* = 2. Listing all scenarios, we conclude: $𝔼[I]=1+\frac{1}{11}(1-Q)$ and 𝔼[U]=2−Q. Using the same method of ratios as Mash/Skmer and solving for *Q*, we obtain:


$$\begin{array}{c} J=\frac{1+\frac{1}{11}(1-Q)}{2-Q}⟹Q=\frac{2J-\frac{12}{11}}{1+J-\frac{12}{11}} \end{array}$$

which adds $-\frac{12}{11}$ to numerator and denominator of the haploid equation. Setting $Q=e^{-\Lambda }≃(1-\theta {)}^{\frac{11}{6}k}$ gives:


$$\begin{array}{c} \hat{\theta }=-\frac{6}{11}\frac{1}{k}\log\left(\frac{2J-\frac{12}{11}}{1+J-\frac{12}{11}}\right)≃1-{\left(\frac{2J-\frac{12}{11}}{1+J-\frac{12}{11}}\right)}^{\frac{6}{11}\frac{1}{k}} \end{array}$$ (4)

This diploid estimate of $\hat{\theta }$ gives a higher value than the haploid estimate (Fig MA in S1 Text) and is only defined for $J>\frac{6}{11}$. To see why, note that under the infinite sites assumption (which breaks at higher distances), two samples with high θ would still have substantial similarity because the two alleles of each individual are sampled from the same pool of alleles available in the population. As shown in Appendix B in S1 Text, a haploid estimate based on Jaccard can be transformed to the diploid estimate using a non-linear equation.

### 4.3 Modeling low coverage and errors

We next study Jaccard for genome skims where some *k*-mers are not covered and others are erroneous. Recall definitions of *L*, *N*, ℓ, ρ, ξ from section 4.1. We henceforth update the definition of $\lambda :=\frac{1}{2}\frac{N}{L}\frac{\ell -k+1}{\ell }$ to be the *haploid k*-mer coverage for a diploid skim, and note that this is half of what we defined before. We aim to model the fact that due to sequencing errors and limited sequencing coverage, a *k*-mer could be missing and/or sampled erroneously in one or both samples, thereby reducing the intersection and potentially increasing the union. Mash deals with errors by requiring each *k*-mer to be observed at least *t* times, noting that for a threshold *t* sufficiently large compared to λ, a *k*-mer sampled *t* times is likely to be error-free. This approach only works for high coverage, since for low coverage, very few *k*-mers will survive this filter. Our approach is to select a t≥1 based on our es*t*imate of coverage. In practice, we set $t=\lfloor \frac{\xi }{2}\rfloor +1$ by default, allowing each sample *j* to have i*t*s own $t_{j}$. We assume that no erroneous *k*-mer will match any alleles in the genome. Let


$$\begin{array}{c} \psi_{j}:=𝔼[\text{number of erroneous kmers per position appearing at least }t_{j}\text{ times in sample }j]\;. \end{array}$$

Thus, each position in expectation adds $\psi_{1}+\psi_{2}$ erroneous *k*-mers to the union and none to the intersection. Since errors are mostly random, with our choice of $t_{j}$, it is safe to assume $\psi_{j}=0$ for $t_{j}>1$ and $\psi_{j}=2\lambda (1-\rho )$ for $t_{j}=1$. We now focus on the error-free *k*-mers, which may be homozygous or heterozygous. Let


$$\begin{array}{c} \eta_{j}(i):=\mathrm{Pr}[\text{sample }j\text{ includes at least }t_{j}\text{ copies of a }k\text{-mer present }i\text{ times in the genome}]=1-F_{\text{P}}(t_{j}-1;i\xi ) \end{array}$$

where *F*_P_ is the CDF of Poisson, which can be calculated using the regularized incomplete gamma function. Since a *k*-mer may be homozygous or heterozygous (effectively covered twice), we are interested in i∈{1,2}. For each location, and for each genotype (Fig 6), we can compute their expected contribution to *U* and *I*, as listed below, from which 𝔼[I]=𝐏·𝐈 and $𝔼[U]=𝐏\cdot 𝐔+\psi_{1}+\psi_{2}$ can be calculated (see Eqs (E) and (F) in S1 Text).


$$\begin{array}{c} \begin{array}{cccc} \text{Genotype g} & 𝐏 & 𝐈=𝔼[I|g] & 𝐔=𝔼[U|g,\text{error-free}]\\ {}[3pt]00\;00 & Q & \eta_{1}(2)\eta_{2}(2) & \eta_{1}(2)+\eta_{2}(2)-\eta_{1}(2)\eta_{2}(2)\\ {}[3pt]00\;01 & \frac{4P}{11} & \eta_{1}(2)\eta_{2}(1) & \eta_{1}(2)+\eta_{2}(1)-\eta_{1}(2)\eta_{2}(1)+\eta_{2}(1)\\ {}[3pt]10\;00 & \frac{4P}{11} & \eta_{1}(1)\eta_{2}(2) & \eta_{1}(1)+\eta_{2}(2)-\eta_{1}(1)\eta_{2}(2)+\eta_{1}(1)\\ {}[3pt]01\;01 & \frac{2P}{11} & 2\eta_{1}(1)\eta_{2}(1) & 2(\eta_{1}(1)+\eta_{2}(1)-\eta_{1}(1)\eta_{2}(1))\\ {}[3pt]11\;00\text{ or }00\;11 & \frac{P}{11} & 0 & \eta_{1}(2)+\eta_{2}(2) \end{array} \end{array}$$

For example, for the genotype 00 01, *I* = 1 if and only if the *k*-mer is sampled at least *t*_1_ times from the first skim where it is homozygous, and *t*_2_ from the second skim, where it only appears in one chromosome; otherwise, *I* = 0. Therefore, $𝔼[I|00\;01]=\eta_{1}(2)\eta_{2}(1)$. For union *U*, we have two distinct *k*-mers. Allele 0 is sampled at least *t*_1_ and *t*_2_ times withou*t* error with probabilities $\eta_{1}(2)$ and $\eta_{2}(1)$ in each individual and $\eta_{1}(2)\eta_{2}(1)$ in both, and allele 1 with probability $\eta_{2}(1)$ in the second individual. Thus, $𝔼[U|00\;01]=\eta_{1}(2)+\eta_{2}(1)-\eta_{1}(2)\eta_{2}(1)+\eta_{2}(1)$. Other scenarios are similar. Replacing 𝔼[I] and 𝔼[U] from Eqs (E) and (F) in S1 Text in $J=\frac{𝔼\left[I\right]}{𝔼\left[U\right]}$ and solving for Q (ratio estimator) results in:


$$\begin{array}{cc} Q= & 1+11\frac{J(\eta_{1}(2)+\eta_{2}(2))-(1+J)\eta_{1}(2)\eta_{2}(2)+\psi_{1}+\psi_{2}}{6J(2\eta_{1}(1)+2\eta_{2}(1)-\eta_{2}(2)-\eta_{1}(2))+(1+J)(-4(\eta_{1}(1)\eta_{2}(1)+\eta_{1}(1)\eta_{2}(2)+\eta_{1}(2)\eta_{2}(1))+11\eta_{2}(2)\eta_{1}(2))}. \end{array}$$ (5)

### 4.4 Parameter estimation for diploid skims

To compute Eq (5), we need to estimate parameters λ,ϵ which immediately give us ξ,η,ψ. Following Skmer, we compute these from the *k*-mer repeat spectrum of each sample, but we model heterozygosity. Let $M_{i}$ denote the number of *k*-mers observed exactly *i* times and assume all errors fall in *M*_1_. Let


$$\begin{array}{c} ℳ:=\text{the set of all unique kmers in a diploid genome of length }L \end{array}$$

$$\begin{array}{c} A_{x,i}:=\text{the event that a genomic kmer}x\text{appears}i\text{times error-free in a sample}. \end{array}$$

Let M:=|ℳ| and note M≥L and $𝔼[M]=L(2-(1-\theta {)}^{k})$ (Appendix A in S1 Text). Clearly, we need to estimate θ for each sample in order to estimate genome length and coverage. Let As shown in Eq (A) in S1 Text, for *i* > 1,


$$\begin{array}{c} 𝔼\left[M_{i}\right]=\mathrm{Pr}(A_{x,i}|x\in ℳ)=\frac{e^{-2\xi }\xi^{i}\left(\left(2^{i}-2e^{\xi }\right)(1-\theta {)}^{k}+2e^{\xi }\right)}{i!\left(2-(1-\theta {)}^{k}\right)}\;. \end{array}$$ (6)

A family of equations can be obtained by taking the ratio of two consecutive instances of Eq (6) for i≥2:


$$\begin{array}{c} \nu_{i}=\frac{𝔼\left[M_{i+1}\right]}{𝔼\left[M_{i}\right]}=\frac{\mathrm{Pr}(A_{x,i+1}|x\in ℳ)}{\mathrm{Pr}(A_{x,i}|x\in ℳ)}=\frac{2\xi }{\left(i+1\right)}\left(\frac{e^{\xi }-\left(e^{\xi }-2^{i}\right)(1-\theta {)}^{k}}{2e^{\xi }+\left(2^{i}-2e^{\xi }\right)(1-\theta {)}^{k}}\right)\;. \end{array}$$ (7)

Using $\nu_{i+1}$ and $\nu_{i}$ enables us to completely eliminate θ (see Eq (C) in S1 Text) and allows us to arrive at an estimate of ξ:


$$\begin{array}{c} \hat{\xi }=\frac{3}{4}(i+1)\nu_{i}-\frac{1}{4}\sqrt{\nu_{i}(i+1)(9(i+1)\nu_{i}-8(i+2)\nu_{i+1})} \end{array}$$ (8)

With $\hat{\xi }$ estimated, we then use Eq (7) to estimate θ directly. It can be confirmed that


$$\begin{array}{c} \hat{\theta }=1-(\frac{2e^{\xi }(\xi -\nu_{i}(1+i))}{\nu_{i}(1+i)(2^{i}-2e^{\xi })+2\xi (e^{\xi }-2^{i})}{)}^{1/k} \end{array}$$ (9)

satisfies (7), giving us an estimator for θ. While any *i* can be used, we follow Skmer [26] and set it to $\text{arg}\;{\text{max}}_{i>1}{\hat{M}}_{i}$ (i.e., the first mode of the skim’s *k*-mer repeat spectrum).

Note that when $9(i+1)\nu_{i}<8(i+2)\nu_{i+1}$, Eq (8) has no solutions, and a different strategy must be used. Let us assume a $\theta^{*}$ is given. Then, we can use Eq (7) directly to estimate ξ by numerically optimizing $\text{arg}\;{\text{min}}_{\xi }(\nu_{i}-\frac{2\xi }{1+i}(\frac{e^{\xi }-(e^{\xi }-2^{i})(1-\theta^{*}{)}^{k}}{2e^{\xi }+(2^{i}-2e^{\xi })(1-\theta^{*}{)}^{k}}){)}^{2}.$ For $\theta^{*}$, we can start with a fixed value (default: $\theta^{*}=0.005$). When multiple individuals are available, we can then use an iterative approach to update $\theta^{*}$. In each pass *r*, we use the pairwise sample distances from the previous round (i.e., $\theta_{r-1}$, as described in section 4.3) as the new $\theta_{r}^{*}$ to recompute coverage and error, which we then use to obtain a new pairwise distance $\theta_{r}$. We continue for a fixed number of passes (default: three), and empirically compare this method to using Equation (9).

Regardless of which method is used to compute $\hat{\xi }$, we next need to decompose it into λ and $\rho =(1-\epsilon {)}^{k}$. To do so, we cannot use the family of equations given by (7) because they are only a function of ξ. Instead, we need to examine error-prone *k*-mers with *i* = 1. Note that $𝔼[\sum_{i=1}^{\infty }iM_{i}]=2L\lambda =2\lambda \frac{𝔼[M]}{2-(1-\theta {)}^{k}}$. Then, since all erroneous *k*-mers are assumed to be in *M*_1_, as shown in Eq (D) in S1 Text, we can derive:


$$\begin{array}{cc} 𝔼[M_{i}] & =\left\{ \begin{array}{cc} 𝔼[M]\frac{e^{-2\xi }\xi^{i}\left(\left(2^{i}-2e^{\xi }\right)(1-\theta {)}^{k}+2e^{\xi }\right)}{i!\left(2-(1-\theta {)}^{k}\right)} & i\geq 2\\ 𝔼[M]\frac{2\left(\lambda -e^{-2\xi }\left(e^{\xi }-1\right)\xi \left((1-\theta {)}^{k}+e^{\xi }\right)\right)}{2-(1-\theta {)}^{k}} & i=1 \end{array}\right.⟹\\ \hat{\lambda } & =\frac{𝔼\left[M_{1}\right]}{𝔼\left[M_{i}\right]}\frac{1}{2i!}\xi^{i}\left(\left(2^{i}e^{-2\xi }-2e^{-\xi }\right)(1-\theta {)}^{k}+2e^{-\xi }\right)+\xi \left(e^{\xi }-1\right)\left(e^{-2\xi }(1-\theta {)}^{k}+e^{-\xi }\right) \end{array}$$

for any *i* > 1. We can then estimate the error rate from $\hat{\lambda }$ and $\hat{\xi }$ as $\hat{\epsilon }=1-(\hat{\xi }/\hat{\lambda }{)}^{1/k}$.

### 4.5 Experimental setup

**Simulations.** To test the accuracy of DipSkmer, we used the coalescent simulator msprime [35] to generate four haploid chromosomes with a range of θ values. To achieve this, we set rates of mutation (μ) and recombination (*r*) to $0.3\times 10^{-8}$ (which is reasonable [47]), and vary effective population sizes ($N_{e}\in \{8.3\times 10^{4},2.5\times 10^{5},\;5\times 10^{5},\;7.5\times 10^{5},\;1\times 10^{6}\}$), which lead to $\theta =4N_{e}\mu$ to be 0.001, 0.003, 0.006, 0.009, or 0.012, resp. We repeat the process 5 times for three genome sizes (15 replicates in total). For each replicate, we divide the four chromosomes into two diploid individuals at random. To test whether DipSkmer can also estimate the divergence between populations, we perform separate msprime simulations with a demography where an initial parent population ($N_{e}=5\times 10^{5}$) splits into two child populations with no change in $N_{e}$ and no migration. Two diploid samples are generated for each of the child populations (eight chromosomes in total). To test different levels of divergence, we adjust the number of generations (τ) back in time when the populations split ($\tau \in \{6.25\times 10^{4},1.25\times 10^{5},2.5\times 10^{5},5\times 10^{5},\;1\times 10^{6},\;2\times 10^{6}\}$). These lead to coalescent unit ($\tau /4N_{e}$) divergences of {0.03125, 0.0625, 0.125, 0.25, 0.5,1}. Note that 1 coalescent unit is high and corresponds to the phylogenetic (rather than population genetic) scale; it is included to test the limits of the methods. Also note that the isolation between populations breaks the random mating assumptions of DipSkmer, and thus, this experiment is meant to test the impacts of model violations.

We generate genomes and genome skims for each simulation. To make these realistic in terms of *k*-mer repeat spectra, we replace the parental genome (root) with one of three assemblies with varying *k*-mer repetitiveness (Table D in S1 Text). We impose the genotypes (i.e., VCFs) generated by msprime onto the corresponding sites of the genome, selecting among alternative nucleotides at random and leaving the unmutated sites unchanged. For each resulting genome, we use ART [36] to simulate Illumina short reads (Phred score = 25, ℓ = 150) at coverage c∈{0.5×,1×,2×,4×,8×}, noting that each of the two diploid chromosomes has a coverage of $\frac{c}{2}$. We use the resulting reads as input to DipSkmer, with 1 – 3 passes of $\theta^{*}$ calculations, to DipSkmer with Eq (7) for coverage, and alternative Jaccard-based methods, Mash [23] and Skmer [26]. Variants of DipSkmer differ in the calculation of within-sample θ used to estimate coverage; however, the accuracy is always measured based on the θ obtained from pairs of samples. We measure error as $\frac{\left(\hat{\theta }-\theta \right)}{\theta }$, averaged over all replicates, where $\hat{\theta }$ is the estimated pairwise distance and θ is the true pairwise distance. We also report mean absolute error (MAE), defined as $\frac{|\hat{\theta }-\theta |}{\theta }$. We measure the true distance as the percentage of substituted positions in the simulated genome. For population differentiation experiments, we estimate $D_{xy}$ (average heterozygosity between pairs of samples taken from different populations) and Weir and Cockerham’s $F_{st}=1-\frac{\theta_{x}+\theta_{y}}{2D_{xy}}$ [37], comparing estimated values to the ground truth, calculated based on simulated genomes.

**Biological data.** To test DipSkmer on empirical data, since true θ values are not known, we compare ReSkmer, DipSkmer, Skmer, and Mash to previously published [38] θ estimates obtained by ANGSD [39], which is a widely used method based on read mapping, genotyping, and computing Site Frequency Spectrum (SFS). We analyze a dataset [38] consisting of 12 total populations across six different species with a range of θ between 0.0006−0.0068 (Table B in S1 Text). Because high-quality reference genomes are not available for all analyzed species, we use RESPECT to estimate the genome repeat spectra required by ReSkmer. Output using this method is referred to as ReSkmer-noref. Beyond the difference between θ values, we also measure the Pearson correlation of our estimates of θ to those produced by ANGSD. To reduce the impacts of contamination on alignment-free methods [48], we utilize a published protocol [40], which includes the removal of adapters and deduplication of reads using BBtools [49]. Bacterial and archaeal reads are then removed using CONSULT-II [50], and human reads are filtered out using Kraken2 [51]. To test methods with low-coverage skims, we use RESPECT [44] to estimate genome size, then subsample the coverage of all genome skims to target coverages c∈{0.5×,1×,2×,4×,6×,8×}. Note that two populations (Cocos island and Cristobal) were not sequenced deep enough to reach 8× coverage and are absent from high coverage Pearson correlations.

To benchmark DipSkmer’s ability to measure population structure in an empirical dataset, we analyze a short-read WGS dataset of three *Sillago sinica* populations (Wenzhou, Qingdao, Dongying) [52]. To prepare the sequencing reads for ANGSD [39], we first performed adapter and quality trimming using BBtools [49] and used BWA-MEM [53] to map reads to a publicly available reference assembly [54]. We then use ANGSD to obtain an IBS (identity by state) matrix, run a principal coordinate analysis (PCoA) on it, and analyze the first two components. To judge the ability of reference-free methods to obtain population structure, we first used Skmer’s built-in subsampling approach [55] to generate 100 replicates of reads subsampled from 4× genome skims. We then use Skmer, ReSkmer, and DipSkmer, to construct distance matrices based on those subsamples. Here we compare against ReSkmer’s two modes. ReSkmer-ref is the mode that uses the genome repeat spectra obtained from a reference assembly; meanwhile, ReSkmer-noref uses repeat spectra estimated from the genome skim using RESPECT. Next, we use the Procrustes method to align each of these distance matrices to ANGSD’s PCoA and summarize the results by plotting polygons in regions with 33% highest density of points per sample (representing confidence intervals). To quantify the similarity between ANGSD and other methods at a range of coverages, we use the vegan package [56] in R to perform a symmetric Procrustes analysis repeatedly (a statistical test referred to as “protest”) to obtain *t*_0_, a statistic derived from *t*he sum of squares ($t_{0}=1-ss$) between rotated matrices. This tells us how similar projections from reference-free methods are to those produced by ANGSD; *t*_0_ ranges from 0 (dissimilar) to 1 (identical).

To get $F_{st}$ values with the reference-free methods, we take the distance matrices output by each method and compute Weir and Cockerham’s (WC) definition of $F_{st}$[37] ($F_{st}=1-\frac{\theta_{x}+\theta_{y}}{2D_{xy}}$). To get $F_{st}$ with ANGSD, we use two different approaches. First, we take the distances from the IBS matrix to compute WC $F_{st}$ (referred to as “ANGSD $F_{st}$ (IBS)”). Second, we use ANGSD and realSFS to generate the 2D Site Frequency Spectra (2D SFS) between all pairs of populations and obtain the unweighted Reynolds’ $F_{st}$ [57].

## Supporting information

S1 Text Appendices A-B, Tables A-D, Figs A-M. (PDF)
